# Sacral neuromodulation for overactive bladder using the InterStim and BetterStim systems

**DOI:** 10.1038/s41598-022-26267-y

**Published:** 2022-12-24

**Authors:** Lingfeng Meng, Zijian Tian, Yaoguang Zhang, Jianye Wang, Limin Liao, Guoqing Chen, Xiaojun Tian, Lulin Ma, Yan Li, Benkang Shi, Yong Zhang, Qing Ling, Peng Zhang, Zhongqing Wei, Tie Zhong, Zhihui Xu, Jiayi Li, Deyi Luo

**Affiliations:** 1 Department of Urology, Beijing Hospital, National Center of Gerontology, Institute of Geriatric Medicine, Chinese Academy of Medical Sciences, No. 1 Da Hua Road, Dong Dan, Beijing, 100730 China; 2 Beijing Hospital Continence Center, Beijing, China; 3 Department of Urology, China Rehabilitation Research Centre, Rehabilitation School of Capital Medical University, Beijing, China; 4 Department of Urology, Peking University Third Hospital, Beijing, China; 5 Department of Urology, Qilu Hospital, Shandong University, Jinan, China; 6 Department of Urology, Beijing Tiantan Hospital, Capital Medical University, Beijing, China; 7 Department of Urology, Tongji Hospital, Huazhong University of Science and Technology, Wuhan, China; 8 Department of Urology, Beijing Chaoyang Hospital, Institute of Urology, Capital Medical University, Beijing, China; 9 Department of Urology, The Second Affiliated Hospital of Nanjing Medical University, Nanjing, China; 10 Department of Urology, The Second Affiliated Hospital of Xi’an Jiaotong University, Xi’an, China; 11 Department of Urology, Zhejiang Provincial People’s Hospital, Hangzhou, China; 12 Department of Urology, Renji Hospital, Shanghai Jiao Tong University School of Medicine, Shanghai, China; 13 Department of Urology, West China Hospital, Sichuan University, Chengdu, China

**Keywords:** Urology, Bladder, Urological manifestations

## Abstract

This study aimed to evaluate differences in the clinical outcomes of different sacral neuromodulation systems (InterStim and BetterStim) used in the treatment of overactive bladder. Data from a previously established database of sacral neuromodulation in China (the InterStim system) and a 2020 clinical trial of the BetterStim system were screened. Patients with overactive bladder undergoing stage II implanted pulse generator implantation were selected for analysis and divided into InterStim and BetterStim system groups. Voiding diaries and subjective scores obtained preoperatively, after stage I tined-lead implantation (experience period), and after stage II implanted pulse generator implantation were compared between the two groups. This study included 113 patients with overactive bladder (43, InterStim system group; 70, BetterStim system group). Voiding diaries and subjective scores significantly improved in both the InterStim and BetterStim system groups over the treatment period. Specifically, the urination frequency (all P < 0.001), average voiding volume (all P < 0.001), and average urinary leakage (InterStim, P < 0.05; BetterStim, P < 0.01) in both groups significantly improved at different periods during treatment. At the same time, the urgency perception scale (P < 0.001) and OAB-related quality of life score (InterStim, P < 0.05; BetterStim, P < 0.01) also significantly improved. There was no significant difference in urination frequency at baseline between the two groups (P = 0.169). Urination frequency was significantly higher in the BetterStim system group than in the InterStim group during the experience period and at follow-up (P = 0.031, P = 0.006). There was no significant difference in the number of urinary leakages between the different systems at baseline (P = 0.662), although this was higher in the InterStim system group during the experience period (P = 0.016), and the difference disappeared at the last follow-up (P = 0.565). There were significant differences in baseline urgency perception scale (P = 0.001) and OAB-related quality of life score (P < 0.001) between the two groups; however, these differences were not maintained at follow-up (P = 0.81, P = 0.479). Both sacral neuromodulation systems are safe and effective in treating overactive bladder. The InterStim system may be more beneficial for patients with dry overactive bladder. Satisfactory outcomes may be achieved with the BetterStim system in patients with wet overactive bladder. However, further studies are required to confirm this finding.

## Introduction

The International Continence Society has defined overactive bladder (OAB) as a syndrome characterized by urgent urination, often accompanied by frequent urination and nocturia, with or without urgent urinary incontinence (UUI)^1^. Although OAB does not directly affect the life expectancy of patients, it often makes patients feel anxious, depressed, and helpless and has a negative impact on their quality of life.

In China, according to the first large-scale epidemiological survey released by the Chinese Urology Association in June 2010, the overall prevalence of OAB in people aged > 18 years was 5.2%, with an increase in the incidence rate with age. The prevalence of OAB was 11.3% in people aged > 40 years^2^. These figures are expected to increase further as the population ages and the demand for good quality of life increases. According to a survey in 2018, nearly one-fourth of Chinese people aged > 40 years had experienced symptoms of OAB^3^.

Sacral neuromodulation (SNM) has been widely used as a third-line treatment for OAB. Although China performed the first SNM implantation in 1999, the application of SNM remains limited. By 2020, the cumulative number of implants used was only a few hundred^4^. Imported stimulators (Medtronic, InterStim system, United States) for clinical use are expensive, increasing the financial burden on the medical care system and patients and limiting the application of this therapy in China.

In this context, the development of the novel SNM BetterStim system by Beijing PINS Medical Co., LTD. is aimed at reducing the costs of the treatment, such that it benefits more patients in need. At present, its efficacy and safety have been preliminarily confirmed, but the long-term clinical effect still needs to be observed. In addition, compared with the InterStim SNM system, the BetterStim SNM system equipment is more suitable for Chinese body size, and supports remote-programming function^5,6^.

This study aimed to compare the clinical effects of the InterStim and BetterStim systems in treating patients with OAB in order to identify the characteristics of each system, thereby helping clinicians and patients make more informed treatment choices.

## Methods

### Clinical data

The InterStim system data were obtained from the previously established Chinese SNM database. The research design and inclusion and exclusion criteria have been published in detail elsewhere^4,7^. The BetterStim system data were obtained from clinical trials completed in 2020. The primary criteria for inclusion were as follows: (1) age ≥ 16 years; (2) an established refractory OAB diagnosis; (3) normal upper urinary tract function; (4) bladder capacity > 100 mL; (5) willingness to undergo minor surgery; and (6) compliance with follow-up requirements. The main exclusion criteria were as follows: (1) multiple sclerosis, spinal cord injury, stroke, and other neurological diseases; (2) associated uncontrolled urinary tract infection, urinary tract obstruction, or urinary tract tumor; (3) participation in other clinical trials within 3 months of the present trial; and (4) other circumstances that the researcher considered inappropriate for participation in this trial. The stimulator parameters used in this study were all unified standards, that is, the frequency was 14 Hz, the pulse width was 210us, and the amplitude was slightly below the sensory threshold. This study only included patients who underwent stage II permanent implantation, and all the patients provided written informed consent to participate in the study. For participants that are minors, informed consent obtained from their parents and/or legal guardian.

The baseline period was defined as the period before the patients underwent stage I implantation. The experience period was defined as the period after stage I implantation and before stage II permanent implantation. Based on the different implanted systems, the patients were divided into the InterStim and BetterStim system groups. The degree of improvement in the different symptoms between baseline and the experience period and after permanent implantation (at the last follow-up) was compared. The differences in the degree of improvement in the different symptoms were compared between the two groups.

### Evaluation of therapeutic effect

In this study, both subjective and objective indicators were used to evaluate patient response to treatment. Subjective indicators consisted of the scaled scores of patients in different treatment periods, including urgency perception scale (UPS) and OAB-related quality of life (OAB-qol) score^8,9^. Objective indicators reflected the voiding diary of patients 3–5 days before and on the follow-up day. Only the data most closely related to OAB symptoms (daily urination times, average urine volume, and daily urine leakage times) were included in the analysis.

The main outcome of this study was the difference in the improvement of symptoms and scores of patients with OAB between the BetterStim and InterStim systems, and the reasons for these differences were further analyzed. The secondary outcome was the incidence of adverse events associated with the different systems. All clinical data were collected after obtaining study approval from the Ethics Departments of Beijing Hospital (approval number: 2015BJYYEC-08-04). All procedures performed in this study were in accordance with the Declaration of Helsinki (as revised in 2013).

### Statistical analysis

Data analysis was performed using SPSS statistical software (version 22.0, SPSS Inc., Chicago, IL, USA). Repeated measures analysis of variance and Mauchly’s sphericity test were used for analysis of data derived from the same system. Any data that failed to pass the test of sphericity were corrected by Greenhouse–Geisser and/or Huynh–Feldt correction methods. An independent sample t-test was used to compare data derived from the different systems. The chi-square test was used for intergroup comparisons of qualitative data. Statistical significance was set at P < 0.05.

### Ethics declarations

All clinical data were collected after approval of the study by the Ethics Departments of Beijing Hospital (approval number: 2015BJYYEC-08-04). All the patients provided written informed consent to participate in the study. The authors are accountable for all aspects of the work in ensuring that questions related to the accuracy or integrity of any part of the work are appropriately investigated and resolved. All procedures performed in this study were in accordance with the Declaration of Helsinki (as revised in 2013).

## Results

A total of 113 patients with OAB were included in the study. There were 43 and 70 patients in the InterStim and BetterStim system groups, respectively. Baseline information for both groups is shown in Table 1. The proportion of male patients was 34.9% (15/43) in the InterStim system group and 28.6% (20/70) in the BetterStim system group (P > 0.05). The follow-up time was 21.63 ± 12.53 months for the InterStim system group and 33.33 ± 15.59 months for the BetterStim system group (P < 0.001). The baseline data were comparable between the two groups, excluding the longer follow-up time in the BetterStim system group.

**Table 1:** Baseline information.

	System		P value
	InterStim (N = 43), N (%)	BetterStim (N = 70), N (%)	
Male	15 (34.9%)	20 (28.6%)	0.481
Age (years)	52.35 ± 19.92	52.73 ± 15.79	0.938
Follow-up time (months)	21.63 ± 12.53	33.33 ± 15.59	** < 0.001**
Complications	4 (9.3%)	10 (14.3%)	0.435

Significant values are in bold.

Significant improvements were noted in daily urination times, average volume, and daily urine leakage time in both groups at different periods, and no significant differences were noted between the groups in these three variables at baseline (Table 2). It is noteworthy that significantly higher daily urination times were observed during the experience period and at the last follow-up in the BetterStim system group compared to the InterStim group. Significant between-group differences in the daily urine leakage time were observed only during the experience period, and these values were higher in the InterStim system group.

**Table 2:** Objective indicator data.

	System				Between-group analysis (P value)
	N	InterStim	N	BetterStim	
**Daily urination times**					
T0	42	25.26 ± 13.54	70	29.15 ± 14.88	0.169
T1	40	14.20 ± 4.41	70	17.21 ± 9.93	**0.031**
T2	33	13.05 ± 5.16	67	17.65 ± 11.24	**0.006**
Within-group analysis		**F (1.1, 33.8) = 37.381, P < 0.001**		**F (1.7, 114.3) = 56.978, P < 0.001**	
**Average voiding volume (ml)**					
T0	39	79.44 ± 59.74	70	94.71 ± 54.3	0.177
T1	40	149.98 ± 63.06	70	138.21 ± 64.50	0.356
T2	29	146.88 ± 50.63	67	151.45 ± 80.84	0.738
Within-group analysis		**F (1.5, 40.6) = 94.853,** **P < 0.001**		**F (1.7, 111.8) = 38.859,** **P < 0.001**	
**Daily urine leakage times**					
T0	32	2.41 ± 3.30	70	1.98 ± 5.07	0.662
T1	28	1.79 ± 2.46	69	0.52 ± 1.55	**0.016**
T2	25	0.80 ± 1.19	67	0.56 ± 1.94	0.565
Within-group analysis		**F (1.4, 32.5) = 5.243, P < 0.05**		**F (1.3, 85.5) = 6.443, P < 0.01**	

*T0* baseline data, *T1* experience period data, *T2* last follow-up data.

Significant values are in bold.

Patients with OAB using the different systems were satisfied with the improvements obtained in the subjective indicators (Table 3). Specifically, UPS and OAB-qol scores significantly improved over the treatment period in both groups. Regarding between-group differences, UPS and OAB-qol scores were significantly lower in the BetterStim system group at baseline, but these differences were not maintained at the last follow-up.

**Table 3:** Subjective indicator data.

	System				Between-group analysis (P value)
	N	InterStim	N	BetterStim	
**UPS**					
T0	43	3.91 ± 1.53	70	2.96 ± 1.37	**0.001**
T1	41	2.02 ± 1.24	70	1.73 ± 1.50	0.259
T2	34	1.91 ± 0.93	67	1.85 ± 1.66	0.81
Within-group analysis		**F (1.7, 55.6) = 78.789, P < 0.001**		**F (2, 132) = 28.47, P < 0.001**	
**OAB-qol score**					
T0	36	3.28 ± 1.89	70	1.58 ± 0.87	** < 0.001**
T1	35	4.03 ± 1.18	69	3.11 ± 1.00	** < 0.001**
T2	33	4.12 ± 1.11	67	3.94 ± 1.24	0.479
Within-group analysis		**F (1.1, 31.9) = 5.175, P < 0.05**		**F (1.7, 115.2) = 96.054, P < 0.001**	

*T0* baseline data, *T1* experience period data, *T2* last follow-up data, *UPS* urgency perception scale, *OAB-qol* overactive bladder related quality of life.

Significant values are in bold.

At the last follow-up, 14 patients experienced device-related adverse events. Of these, four patients were in the InterStim system group, with two cases of recurrent symptoms, one case of disconnection between 0 and 3 o’clock, and one case of drowsiness. With repeated programmed adjustments and avoidance of the faulty circuit, all the patients were able to maintain curative efficacy without surgical intervention. Device-related adverse events occurred in 10 patients in the BetterStim system group, including undesirable changes in stimulation in 10 patients, efficacy fluctuations in eight patients, and pain at the implant site in five patients. A total of eight patients removed the device due to adverse events after stage II permanent implantation, including four cases of unsatisfactory or ineffective treatment effect, one case of discomfort at the implantation site, one case of wire breakage, one case of capsular exudate, and one case requiring cystectomy. There was no significant difference in the incidence of complications between the two groups (P = 0.435).

## Discussion

To the best of our knowledge, this study is the first to compare the differences in the clinical outcomes of two SNM systems (InterStim and BetterStim systems) in the treatment of OAB. This comparison provides reference for clinicians in clinical treatment selection.

As an advanced, innovative, and minimally invasive treatment method, SNM has been recommended by several professional international society guidelines for the treatment of refractory lower urinary tract dysfunction diseases, including OAB and UUI, and is used worldwide^10^. The budget spent by the United States on UUI in 2020 exceeded 80 billion dollars^11^. Specifically, for each patient with OAB, the cost is approximately $11,134 per year, which represents a considerable socio-economic burden^12^. As a developing country with a population of 1.44 billion, there are more patients with OAB in China than in any other country. Unfortunately, compared with China’s average annual income of 60,000 yuan, the imported SNM, which costs as much as 130,000 yuan, is undoubtedly a “luxury” treatment and has not yet been included in China’s latest medical insurance catalog (2020 edition). On this basis, a domestic stimulator was developed and successfully completed a clinical trial in 2020^5,6^.

This analysis confirmed the effective and stable performance of both systems. The improvements in voiding diaries and subjective scores observed in both group patients are comparable to those previously reported in patients using other types of SNM devices^13^. Comparing the two systems directly, we found that there were slight differences concerning improvements in daily urination and urine leakage times. Specifically, although no significant difference was observed between the two groups at baseline, daily urination times were significantly higher in the BetterStim system group than the InterStim group during the experience period and at the last follow-up. Differences in daily leakage times were noted only in the experience period, with significantly higher daily leakage times in the InterStim system group than the BetterStim group.

These findings appear contradictory; however, a more refined classification method for OAB, namely dry and wet OAB, is required. UUI is a common symptom of OAB. According to the current definition of OAB by the International Continence Society, all patients with UUI have OAB, but not all patients with OAB have UUI^1^. In fact, up to 40–50% of women and 80% of men with OAB do not have UUI; this condition is termed dry OAB^14,15^. Conversely, OAB associated with UUI is referred to as wet OAB. Data from this study suggest that the InterStim system is more responsive during the experience period and may be more beneficial for patients with dry OAB. At the same time, data analysis demonstrated better efficacy of the InterStim system at the last follow-up. However, in this study, the follow-up time was longer in the BetterStim system group; therefore, it was not possible to conclude that the InterStim system had better efficacy in the mid-term. For patients with wet OAB, the BetterStim system showed better performance during the experience period, thereby possibly increasing the conversion rates. However, the above conclusions are based on the interpretation and analysis of the data included in this study based on clinical experience, and further studies are needed to verify and evaluate the preliminary conclusions of this study.

The differences in subjective scores between the different systems were analyzed, with significant differences in the UPS and OAB-qol scores observed at baseline. Specifically, patients in the InterStim system group had a stronger sense of urgency, and there was a greater impact on the OAB-qol of patients in the BetterStim system group. These differences disappeared at the last follow-up visit. In our view, the main reason for this finding is financial. The InterStim system is available for installation at the patient’s own expense, whereas the BetterStim system is available after screening as part of a clinical trial at no cost to the patient. Therefore, it is understandable that patients who chose self-paid InterStim implants reported greater subjective urgency, whereas baseline data in the BetterStim patients showed a greater impact on OAB-qol. We speculate that the effect of clinical trial recruitment led to a lower OAB- qol score in the BetterStim group during screening. In addition, for patients in clinical trials, there is a dedicated resident who is responsible for regular follow-up via telephone and adjustment of the stimulation parameters according to the needs of patients during each follow-up visit^5^. Overall, participants in clinical trials received more attention than patients undergoing routine clinical care, which may have led to a greater improvement in OAB-qol scores in the BetterStim system group.

The reported complication rates vary widely among studies, systems, and devices. In a randomized controlled trial of the InterStim system, during the 12-month follow-up, Noblett et al. found that approximately 30% (82/272) of patients showed device-related adverse events, although in most cases, surgical intervention was not required^16^. This incidence was significantly higher than the incidence of complications noted in the InterStim system group in our study. We postulate that the reason for this disparity is that the sample size of the InterStim system group in this study was small. Additional studies with larger sample sizes are required to assess the validity of these results. During the 2-year follow-up of a new rechargeable SNM device (Axonics System), only 16% (13/51) of the patients had adverse reactions^17^. This ratio is similar to the complication rate of the BetterStim system group in this study, and both are clinical trials, suggesting that adequate patient education and management may play a positive role in reducing the incidence of adverse events.

This study has several limitations. First, although more than 110 patients with OAB undergoing SNM were included in this study, the sample size may have been insufficient. The test efficiency was reduced after grouping according to the different systems, which may have affected the accuracy of our conclusions. Second, relatively few research indicators were used in this study. Since we used patients from two different datasets, only the corresponding indicators were included in this study. Third, the details of the mechanisms underlying these findings have not been clarified. The working principle of the two systems is the same, with electrical pulses applied to specific sacral nerves; however, the analysis shows that the clinical effects are slightly different.

The use of SNMs in China is increasing every year; therefore, it is necessary to carefully evaluate this therapy. To the best of our knowledge, this study is the first to compare the clinical outcomes of two different SNM systems used in the treatment of OAB. This study demonstrated that the InterStim system was more effective during the experience period and may bring greater benefit to patients with dry OAB. However, for patients with wet OAB, the BetterStim system performed better during the experience period and may help to further increase the conversion rates. Further studies are required to confirm is one system is more efficient for one subtype of OAB to improve the choice of treatment based on individualized characteristics.

## Data Availability

The datasets generated and/or analyzed during the current study are not publicly available due to the confidentiality of clinical trial data but are available from the corresponding author on reasonable request.

## References

[1] Abrams P (2003). The standardisation of terminology in lower urinary tract function: Report from the Standardisation Sub-Committee of the International Continence Society. Urology.

[2] Wang Y (2011). Prevalence, risk factors, and impact on health-related quality of life of overactive bladder in China. Neurourol. Urodyn..

[3] Wang JY, Liao L, Liu M, Sumarsono B, Cong M (2018). Epidemiology of lower urinary tract symptoms in a cross-sectional, population-based study: The status in China. Medicine.

[4] Meng L (2020). Influence of patient sex on the effectiveness of sacral neuromodulation: A cohort study from China. Int. J. Surg..

[5] Zhang Y (2021). Intermediate-term results of a prospective, multicenter study on remote programming sacral neuromodulation for refractory overactive bladder. Transl. Androl. Urol..

[6] Zhang Y (2019). Remotely programmed sacral neuromodulation for the treatment of patients with refractory overactive bladder: A prospective randomized controlled trial evaluating the safety and efficacy of a novel sacral neuromodulation device. World J. Urol..

[7] Meng L (2020). Analysis of the correlation between the clinical effect of sacral neuromodulation and patient age: A retrospective multicenter study in China. Neuromodulation.

[8] Cardozo L, Coyne KS, Versi E (2005). Validation of the urgency perception scale. BJU Int..

[9] Coyne K, Revicki D, Hunt T (2002). Psychometric validation of an overactive bladder symptom and health-related quality of life questionnaire: The OAB-q. Qual. Life Res..

[10] Goldman HB (2018). International Continence Society best practice statement for use of sacral neuromodulation. Neurourol. Urodyn..

[11] Coyne KS (2014). Economic burden of urgency urinary incontinence in the United States: A systematic review. J. Manag. Care Pharm..

[12] Powell LC, Szabo SM, Walker D, Gooch K (2018). The economic burden of overactive bladder in the United States: A systematic literature review. Neurourol. Urodyn..

[13] Blok B (2020). Two-year safety and efficacy outcomes for the treatment of overactive bladder using a long-lived rechargeable sacral neuromodulation system. Neurourol. Urodyn..

[14] Abrams P, Swift S (2005). Solifenacin Is effective for the treatment of OAB dry patients: A pooled analysis. Eur. Urol..

[15] Milsom I (2001). How widespread are the symptoms of an overactive bladder and how are they managed? A population-based prevalence study. BJU Int..

[16] Noblett K, Benson K, Kreder K (2017). Detailed analysis of adverse events and surgical interventions in a large prospective trial of sacral neuromodulation therapy for overactive bladder patients. Neurourol. Urodyn..

[17] Pezzella A (2021). Two-year outcomes of the ARTISAN-SNM study for the treatment of urinary urgency incontinence using the Axonics rechargeable sacral neuromodulation system. Neurourol. Urodyn..

